# Is active travel associated with greater physical activity? The contribution of commuting and non-commuting active travel to total physical activity in adults^☆^

**DOI:** 10.1016/j.ypmed.2012.06.028

**Published:** 2012-09

**Authors:** Shannon Sahlqvist, Yena Song, David Ogilvie

**Affiliations:** a Medical Research Council Epidemiology Unit and UKCRC Centre for Diet and Activity Research (CEDAR), Institute of Public Health, Cambridge, United Kingdom; b Centre for Physical Activity and Nutrition Research (C-PAN) School of Exercise and Nutrition Sciences, Deakin University, Geelong, Australia; c Transportation Research Group, Faculty of Engineering and the Environment, University of Southampton, United Kingdom

**Keywords:** Physical activity, Walking, Cycling, Transportation, Utilitarian activity

## Abstract

**Background:**

To complement findings that active travel reduces the risk of morbidity and mortality from chronic diseases, an understanding of the mechanisms through which active travel may lead to improved health is required.

**Purpose:**

The aim of this study is to examine the descriptive epidemiology of all active travel and its associations with recreational and total physical activity in a sample of adults in the UK.

**Methods:**

In April 2010, data were collected from 3516 adults as part of the baseline survey for the iConnect study in the UK. Travel and recreational physical activity were assessed using detailed seven-day recall instruments. Linear regression analyses, controlling for demographic characteristics, examined associations between active travel, defined as any walking and cycling for transport, and recreational and total physical activity.

**Results:**

65% of respondents (mean age 50.5 years) reported some form of active travel, accumulating an average of 195 min/week (standard deviation = 188.6). There were no differences in the recreational physical activity levels of respondents by travel mode category. Adults who used active travel did however report significantly higher total physical activity than those who did not.

**Conclusions:**

Substantial physical activity can be accumulated through active travel which also contributes to greater total physical activity.

## Highlights

► Little is known about the association between active travel and physical activity. ► Substantial physical activity can be accrued through active travel. ► Adults who actively travel participate in more overall physical activity.

## Introduction

Walking and cycling for transport provide an opportunity to incorporate frequent physical activity into daily living, with active commuting particularly associated with reductions in morbidity and mortality even after controlling for participation in other forms of physical activity (Gordon-Larsen et al., 2009; Hamer and Chida, 2008; Lindström, 2008; Wen et al., 2006; Wen and Rissel, 2008). To better understand the mechanisms through which active travel may benefit health, detailed examination of the nature of active travel is required, in particular to understand whether those who participate in active travel actually participate in greater physical activity overall (Shepard, 2008).

Small-scale studies suggest that active commuting is associated with greater total physical activity in adults. A study of university students (n = 50) found that those who cycled to their university accumulated more physical activity than those who travelled there by car (Sisson and Tudor-Locke, 2008). In another study, train commuters (n = 177) accumulated 30% more steps per day than car commuters (Wener and Evans, 2007). While these findings are encouraging they need to be substantiated in larger, more representative populations, using a more inclusive measure of active travel (e.g. one that includes walking, cycling and the active component of public transport journeys). Moreover, while previous research has focused on commuting, data from the National Travel Survey (NTS) suggest that only 15% of journeys in the UK are for commuting purposes (Department for Transport, 2009). The aim of this study therefore was to examine the descriptive epidemiology of *all* active travel and its associations with recreational and total physical activity in a sample of adults in the UK.

## Methods

### Sample and study design

These analyses use cross-sectional data from iConnect, a study assessing the impact of infrastructural improvements on travel and physical activity behaviour and carbon emissions. The core module of the iConnect study involves data collected in three areas of the UK: Cardiff, Kenilworth and Southampton. Full details of the evaluation framework, study methods and survey instrument used have been reported elsewhere (Ogilvie et al., 2012, 2011).

Briefly, 22,500 adults living in the study areas in April 2010 were randomly selected from the edited electoral register and were sent a forewarning postcard. A week later, a survey pack containing a letter of invitation, the questionnaire and a consent form were mailed to participants, who were asked to return the completed questionnaire along with the consent form in a reply paid envelope. If a questionnaire had not been returned within two weeks, participants were sent a second survey pack. Upon receipt of their questionnaire, respondents were entered into a prize draw to win one of twenty £25 gift vouchers. 3516 responded, giving a response rate of 15.6%. Ethical approval was obtained from the University of Southampton Ethics Committee (CEE 200809‐15).

### Exposure measure

Travel was assessed using a detailed seven-day recall instrument. For five journey purposes (to and from work; in the course of business; to and from a place of study; for shopping and personal business; and for visiting friends or relatives or other social activities) respondents were asked to recall the number of journeys made and the total time (min/week) spent and the distance (miles/week) travelled using *each* mode (walking, cycling, bus, train, car and ‘other’). The instrument instructed participants to report all modes used as part of the journeys and was therefore able to capture active travel as part of longer journeys (e.g. by public transport). Where a journey involved multiple purposes, participants were asked to report it under the ‘main purpose’. The questionnaire has previously been published in full (Ogilvie et al., 2012).

Where respondents (n = 205) reported the distance travelled but not the time, a time was imputed using the mean observed speed for each mode/purpose combination. Excluding these respondents by way of sensitivity analysis made no substantive difference on the findings (results not shown). For each purpose, travel modes were aggregated into active (walking or cycling) and motorized (bus, train or car). The instrument enabled the walking and cycling done as part of public transport journeys to be distinguished from the public transport stages of those journeys. For this reason, public transport was categorised as ‘motorized’ and the walking and cycling that was recorded as part of the journey as ‘active’. ‘Other’ travel modes which included, for example, taxi, van, caravan or ferry were categorised as motorized.

Measures of commuting and non-commuting active travel were derived. Commuting travel was defined as travel between home and a place of work or study and non-commuting travel as travel for shopping and personal business or for visiting friends/relatives or other social activities. Travel in the course of business was not included in these aggregate measures. Total time (min/week) spent walking and cycling and in all active travel was calculated for commuting and non-commuting travel. Summary travel mode categories were derived according to whether participants reported using only motorized modes (‘motorized’), both active and motorized modes (‘combination’), or only active modes (‘active’) for their commuting travel, their non-commuting travel and for all their travel.

### Outcome measure

Recreational physical activity was assessed using four items adapted from the short form of the International Physical Activity Questionnaire (IPAQ) (Craig et al., 2003). Respondents were asked to recall the number of sessions and the total time spent walking and cycling for recreation (asked separately) and in both moderate- and vigorous-intensity physical activity in the past seven days. Data cleaning procedures similar to those of the short IPAQ were applied whereby for each activity category data were truncated at 1260 min (21 h/week) and participants who reported greater than 6720 min (16 h/day) were excluded (Craig et al., 2003). *Recreational* physical activity (min/week) was computed by summing the time spent in walking and cycling for recreation and in moderate- and vigorous-intensity activity. *Total* physical activity (min/week) was computed by summing the time spent in active travel and recreational physical activity.

### Sociodemographic and other characteristics

Respondents were categorised according to their sex, age, body mass index (computed from self-reported height and weight), highest educational qualification, employment status, ethnicity, annual household income, housing tenure, household car access and children under 16 years of age in the home. For the 22% of the sample with missing data for one or more sociodemographic variables, multiple imputation (with 5 imputations) was used under the assumption of missing at random.

### Analysis

Analyses were done in October 2011 in STATA/SE 11.0. Linear regression analyses were carried out using recreational and total physical activity (min/week) as the dependent variables and travel mode (motorized, combination, active) as the independent variable. Analyses were stratified by commuting status (yes/no) and in commuters, separate models examined associations with travel mode based on commuting, non-commuting and overall travel. The motorized travel category was set as the reference and the lincom command was used to examine differences between those categorised as using a combination of modes and those using only active modes. To examine the possible moderating effect of commuting behaviour, linear regression was also conducted using the entire sample including commuting status as an interaction term. Finally, to examine a possible dose–response relationship linear regression analyses were run with time spent in active travel (categorised as none, < 60 min/week, 60 to 150 min/week and ≥ 150 min/week) as the independent variable and total physical activity as the dependent variable. All models were run with and without adjustment for all sociodemographic variables.

## Results

Of the 3516 respondents, 177 either provided insufficient travel or physical activity data (n = 141) or reported more than three hours of active travel per day (n = 36) and were excluded. Respondents had a mean age of 50.5 years and just over half were female (Table 1). Respondents were more likely to have a university qualification, own their own home or be retired, and were less likely to be aged 18–29 years or have no access to a car according to 2001 census data (Table 1).

2267 respondents reported travel for commuting purposes and 3184 reported travel for non-commuting purposes (Table 2). 46 respondents did not report any travel in the previous week. Overall, 2161 (64.7%) respondents reported some form of active travel; 62.3% (n = 2081) reported walking for transport compared with 11.5% (n = 383) who cycled. Those who cycled spent a mean of 145.5 min/week (SD = 135.4) doing so, while those who walked reported a mean of 176.1 min/week (SD = 176.6) doing so.

### Association between travel mode category and recreational and total physical activity

The time (min/week) spent in recreational and total physical activity by travel mode category (motorized, combination, active) is presented in Table 3. In commuters, there was no statistically significant difference in recreational physical activity by travel mode category. In non-commuters those who reported using a combination of modes accrued an additional 100 min/week of recreational physical activity on average compared with those who only used motorized modes, although this difference was not statistically significant.

Participants who used a combination of modes and those who used only active modes reported more total physical activity than those who used no active modes (Table 3). In commuters this equated to an additional 320.9 min/week (or 46 min/day) for those who used active modes and an additional 189.2 min/week (or 27 min/day) for those who used both active and motorized modes. The associations were statistically significant in adjusted models (Active: ß = 280.1, 95% CI 197.9 to 362.2; Combination: ß = 184.6, 95% CI 150.5 to 218.7, Table 4). Those who used only active modes were also significantly more active than those who used both active and motorized modes (ß = 85.4, 95% CI 18.3 to 172.6). Similar patterns of association were observed with travel mode categories based on commuting and non-commuting travel separately.

Similarly, for non-commuters, those who used only active modes reported an additional 279.4 min/week (or 40 min/day) and those using a combination, an additional 287.1 min/week (or 41 min/day) of total physical activity compared with those who only used motorized modes (see Table 3). These differences were statistically significant in adjusted models (Active: ß = 266.0, 95% CI 132.9 to 399.1; Combination: ß = 241.7, 95% CI 186.8 to 296.6; see Table 4). The total physical activity of non-commuters who used only active modes was not significantly different from those who used both active and motorized modes (ß = 24.3, 95% CI − 106.4 to 155.0).

There was no evidence that commuting status moderated the overall association between travel mode category and total physical activity (ß for interaction = − 118.8, 95% CI − 18.3 to 255.8). There was, however, evidence of an interaction in those who used a combination of modes, in whom the association between travel mode category and total physical activity was weaker in commuters than in non-commuters (ß = − 147.01, 95% CI = − 255.9 to − 8.1).

### Association between quantity of active travel and total physical activity

There was evidence of a dose–response relationship between active travel and total physical activity such that respondents who reported ≥ 60 min/week of active travel participated in significantly more total physical activity than those who reported no active travel (Table 5). This relationship was maintained when examining the association separately for those who used only active modes and for those who used a combination of modes (results not shown).

## Discussion

These findings suggest that adults who walk or cycle for transport accumulate more total physical activity than those who travel using only motorized modes. The findings extend previous studies which have shown associations between commuter cycling and physical activity (Sisson and Tudor-Locke, 2008) and between public transport use and step counts (Wener and Evans, 2007) and physical activity energy expenditure (Morabia et al., 2009, 2010), by examining both walking and cycling and non-commuting travel in a large population sample.

63% of respondents reported walking for transport in the previous week compared with 12% who cycled, findings mirrored in the recent seven-day travel diary of the UK NTS in which 63% of respondents had walked, and 14% had cycled, at least once in the last week (Department for Transport, 2009). The average cyclist in the UK NTS reported ‘just under two hours a week’ cycling for commuting purposes, only slightly less than the 142 min/week reported by cyclists in the current study (Department for Transport, 2011). Comparable data on time spent walking were not available.

Not surprisingly, those who reported using only active modes of transport participated in the greatest total physical activity. Encouragingly, however, those who reported active travel in combination with either public transport or car use were significantly more physically active than those who used only motorized modes of transport. We could not ascertain whether those who used both active and motorized modes did so as part of multi-modal journeys (for example by combining walking with public transport on a single trip) or whether different modes were used for different trips. Nonetheless, it seems reasonable to infer that physical activity is accrued when active travel forms part of a multi-modal journey. Evidence of the health benefits of public transport is not new (Besser and Dannenberg, 2005; Morabia et al., 2009, 2010). Public transport use has been shown to result in significantly more energy expenditure when compared with the same journey made by car (Morabia et al., 2009, 2010) and adults who use public transport have been found to spend 19 min/day walking to and from stops (Besser and Dannenberg, 2005). In this study, 85% of those who used both active and motorized modes reported that their active travel was combined with car travel rather than public transport, suggesting that strategies to support multi-modal car journeys, such as the provision of park-and-ride facilities, may also have potential (Goodman et al., 2012).

### Limitations

In the absence of any clear choice of a validated self-report measure, travel behaviour was assessed using a modified seven-day recall instrument and the measure of physical activity was purposively adapted from the IPAQ. While both are established measures of behaviour, their criterion validity when used together and, consequently, the extent to which participants reported the same activity under both recreational and transport sections, remains to be tested. By way of a sensitivity analysis, we compared the values reported for walking and cycling in the 1333 (40%) respondents who reported walking for both transport and recreation and the 222 (7%) who reported cycling for both transport and recreation. We then assumed that 50% of the walking and cycling reported as transport were also reported as recreation, and recalculated the time spent in total physical activity as 0.5 min/week of active travel + min/week of recreational physical activity. This has not really be captured accurately. It is an equation and should read 0.5 × time spent in active travel + time spent in recreational physical activity. This made no substantive difference to the results of the regression models, suggesting that even after accounting for the possibility of double counting the hypothesis that respondents engaging in active travel accumulated more total physical activity remained supported. The summary travel measure also did not allow reporting of individual trips because pilot work suggested that including a detailed trip-based travel recall instrument would have significantly discouraged participation (Sahlqvist et al., 2011; Ogilvie et al., 2012).

Our sample was composed of participants of a higher socioeconomic status on average than the local population from which they were drawn, and their responses to our questionnaire suggested that they were more likely to meet current physical activity recommendations than respondents in the 2008 Health Survey for England (HSE) (men: 75% versus 42%; women: 69% versus 31%) (The Information Centre for Health and Social Care, 2009), although these differences are likely to partially reflect our use of much more detailed, disaggregate measures of travel and recreational physical activity than those used in HSE. While acknowledging these differences, we have no reason to assume that they would bias the *associations* observed in this study. Furthermore, the travel patterns of our respondents are comparable to those of adults who participated in the recent NTS (Department for Transport, 2011). Although the response rate in this study was low, it is comparable to that achieved in other recent population-based surveys implemented for the purpose of evaluating natural experiments (Cummins et al., 2005; Ogilvie et al., 2008) and may reflect a more global downward trend in survey participation (Curtin et al., 2005; Hox and De Leeuw, 1994; Nicolaas, 2004). Finally, as the findings are based on cross-sectional data they cannot be interpreted as showing that an increase in active travel results in a commensurate increase in physical activity.

### Conclusions

Our findings suggest that substantial physical activity can be accrued through active travel and that this may contribute to greater total physical activity. For the full public health impact of active travel interventions to be realised, longitudinal studies examining whether a shift towards active travel is associated with an increase in physical activity are required.

## Conflict of interest statement

The authors declare that there are no conflicts of interest.

## Figures and Tables

**Table 1:** Demographic characteristics of respondents who completed the iConnect baseline survey conducted in the UK in April 2010 and a comparison with population data.

Characteristic	All^a^ n = 3339	Cardiff		Southampton		Kenilworth	
iConnect	Population	iConnect	Population	iConnect	Population
	n = 1075	n = 1058	n = 1206
	Mean (SD)	Mean (SD)		Mean (SD)		Mean (SD)	
BMI (kg/m^2^)	25.6 (4.8)	25.9 (4.7)	n/a	25.4 (5.4)	n/a	25.6 (4.4)	n/a
	% (n)	% (n)	%	% (n)	%	% (n)	%
Sex^b^							
Male	45.2 (1497)	44.3 (470)	48.4	44.0 (461)	50.2	47.1 (566)	49.4
Female	54.8 (1815)	55.7 (582)	51.6	56.1 (588)	49.8	52.9 (635)	50.6
Age^b^							
18–29	16.3 (532)	14.6 (153)	31.3	28.5 (294)	33.6	7.2 (85)	22.3
30–44	20.8 (679)	22.7 (238)	24.9	22.3 (230)	25.3	17.9 (211)	26.9
45–59	27.6 (899)	27.9 (293)	21.2	24.3 (250)	19.2	30.2 (356)	21.2
60–74	26.3 (859)	26.7 (281)	14.0	17.6 (181)	13.4	33.7 (397)	14.0
75 and over	9.0 (292)	8.2 (86)	8.5	7.4 (76)	8.4	11.0 (130)	9.9
Ethnicity^b^							
White	94.9 (3090)	95.7 (1001)	91.6	92.4 (959)	87.0	96.3 (1130)	90.2
Other	5.1 (167)	4.3 (45)	8.4	79 (7.6)	13.0	3.7 (43)	9.8
Education^c^							
University qualification	40.9 (1324)	44.2 (460)	25.1	37.4 (382)	18.8	41.0 (482)	29.0
‘A’ Level	18.0 (581)	14.8 (154)	11.9	21.0 (215)	15.0	18.0 (212)	10.5
GCSE	19.0 (614)	20.2 (210)	30.4	17.5 (179)	33.7	19.2 (225)	31.9
No formal qualification	22.2 (718)	20.8 (216)	32.6	24.1 (246)	32.5	21.8 (256)	28.5
Employment^c^							
Full-time	41.9 (1369)	45.2 (475)	44.6	12.7 (441)	45.5	38.2 (453)	52.8
Part-time	14.0 (457)	14.0 (147)	11.6	11.3 (120)	11.0	16.1 (190)	11.9
Student	6.6 (214)	2.3 (24)	13.8	14.7 (152)	16.1	3.2 (38)	9.7
Retired	27.3 (892)	27.1 (285)	11.7	20.1 (208)	10.6	33.7 (399)	12.9
Home duties	4.3 (138)	5.1 (54)	5.9	3.5 (36)	6.0	4.1 (48)	4.8
Sick, unemployed or other	6.1 (198)	6.2 (65)	13.1	7.5 (77)	10.8	4.7 (56)	7.9
Yearly household income^c^							
≤£20,000	29.9 (826)	28.1 (253)	n/a	36.9 (311)	n/a	25.6 (262)	n/a
£20,001–£40,000	33.0 (912)	32.5 (293)	n/a	33.0 (278)	n/a	33.3 (341)	n/a
>£40,000	37.2 (1029)	39.4 (355)	n/a	30.1 (254)	n/a	41.1 (420)	n/a
Housing tenure^c^							
Owned	76.9 (2490)	79.8 (839)	69.8	59.3 (617)	57.6	87.3 (1034)	73.2
Rented from private landlord	14.7 (481)	11.3 (119)	13.2	28.3 (295)	18.3	5.7 (67)	12.5
Rented from local authority	7.2 (235)	6.9 (72)	16.9	10.1 (105)	24.1	7.2 (235)	14.2
Other	2.1 (70)	2.0 (21)	n/a	2.3 (24)	n/a	2.1 (25)	n/a
Household car access^c^							
None	13.9 (465)	13.2 (142)	29.7	20.1 (212)	30.3	9.2 (111)	19.4
1	40.5 (1350)	42.6 (457)	44.5	44.0 (465)	45.3	36.5 (428)	42.4
2	36.6. (1220)	35.8 (384)	21.3	28.4 (300)	19.6	44.5 (536)	30.6
≥ 3	9.0 (300)	8.5 (91)	4.5	7.6 (80)	4.8	10.7 (129)	7.6
Children < 16 years of age at home^c^							
Yes	20.6 (679)	21.4 (227)	n/a	19.1 (199)	n/a	21.3 (253)	n/a
No	79.4 (2613)	78.6 (833)	n/a	80.9 (845)	n/a	78.7 (935)	n/a

n/a: comparable population data not available.

a Numbers do not sum up to a total due to missing responses.

b The 2009 population estimation data provided by neighbourhood statistics were used to construct the population profiles except for Cardiff's ethnicity, for which 2001 census data were the only available data (Office of National Statistics, accessed 30.04.12).

c The 2001 census data provided by neighbourhood statistics were used to construct the population profiles (Office of National Statistics, accessed 30.04.12).

**Table 2:** Time (min/week) spent walking and cycling and in all active travel by purpose for respondents of the iConnect survey (n = 3339) conducted in the UK in April 2010.

	n^d^	Participants reporting the behaviour^a^		All participants reporting the purpose^b^			All participants^c^		
Mean (SD)	Median (IQR)	%^e^	Mean (SD)	Median (IQR)	%^f^	Mean (SD)	Median (IQR)
Cycling									
Commuting n = 2267	237	142.1 (124.1)	120 (60–180)	10.5	14.9 (59.1)	0 (0–0)	7.1	10.1 (49.2)	0 (0–0)
Non-commuting n = 3184	249	88.6 (94.3)	60 (30–120)	7.8	6.9 (35.5)	0 (0–0)	7.5	6.6 (34.7)	0 (0–0)
Walking									
Commuting n = 2267	940	134.7 (129.7)	100 (45–180)	41.5	55.8 (106.7)	0 (0–60)	28.2	37.9 (91.7)	0 (0–25)
Non-commuting n = 3184	1810	132.6 (141.2)	90 (40–180)	56.8	75.4 (125.1)	25 (0–100)	54.2	71.9 (123.2)	20 (0–90)
All active travel									
Commuting n = 2267	1059	151.3 (138.9)	120 (60–200)	46.7	70.7 (121.3)	0 (0–100)	31.7	48.0 (105.2)	0 (0–50)
Non-commuting n = 3184	1872	140.0 (148.5)	90 (40–180)	58.8	82.3 (133.1)	30 (0–120)	56.1	78.5 (131.1)	20 (0–110)

a Descriptive statistics restricted to those participants who reported travelling in that way (i.e. cycling for commuting purposes).

b Descriptive statistics restricted to those participants who reported travelling for that purpose (commuting; n = 2267; non-commuting; n = 3184).

c Descriptive statistics for all participants (n = 3339).

d Represents the number of participants who reported travelling in that way.

e Calculated as the number of participants who reported travelling in that way divided by the total number of participants who travelled for that purpose.

f Calculated as the number of participants who reported travelling in that way divided by all the participants.

**Table 3:** Time (min/week) spent in transport-related activity and total physical activity by travel mode category and purpose for respondents (n = 3339) of the iConnect survey conducted in the UK in April 2010.

	Commuters (n = 2267)												Non-commuters (n = 1072)			
Non-commuting travel				Commuting travel				All travel				All travel			
n (%)	Travel^a^	Rec^b^	Total^c^	n (%)	Travel	Rec	Total	n (%)	Travel	Rec	Total	n (%)	Travel	Rec	Total
Motorized	1019 (44.9)	37.6 (92.6)	261.9 (305.9)	299.4 (321.5)	1208 (53.3)	37.5 (74.2)	281.6 (314.4)	319.2 (331.6)	730 (32.2)	n/a	272.0 (319.1)	272.0 (319.2)	448 (41.8)	n/a	260.7 (381.6)	260.7 (381.6)
Combination	1026 (45.3)	194.3 (186.9)	284.1 (303.5)	478.4 (381.0)	701 (30.9)	221.5 (203.4)	262.6 (301.0)	484.2 (401.1)	142.8 (63.0)	187.0 (185.8)	274.2 (301.4)	461.2 (380.1)	574 (53.5)	185.5 (177.7)	362.3 (385.2)	547.8 (451.0)
Active	222 (9.8)	294.1 (230.1)	272.5 (342.9)	566.2 (452.4)	358 (16.0)	285.7 (202.9)	264.0 (303.7)	549.8 (395.3)	109 (4.9)	329.6 (206.0)	263.3 (333.7)	592.9 (433.2)	50 (4.6)	257.7 (228.9)	282.5 (345.6)	540.1 (506.5)

a Mean (SD) time spent in active travel defined as walking and cycling for transport.

b Mean (SD) time spent in recreational physical activity defined as walking and cycling for recreation and other moderate- and vigorous-intensity recreational physical activity.

c Mean (SD) time spent in total physical activity defined as the sum of active travel and recreational physical activity.

**Table 4:** Linear regression examining the association between travel mode category and overall physical activity stratified by commuting status in respondents (n = 3339) of the iConnect survey conducted in the UK in April 2010.

	Commuters (n = 2267)						Non-commuters (n = 1072)	
Commuting travel		Non-commuting travel		Overall travel		Overall travel	
ß^b^ (95% CI)	ß^c^ (95% CI)	ß^b^ (95% CI)	ß^c^ (95% CI)	ß^b^ (95% CI)	ß^c^ (95% CI)	ß^b^ (95% CI)	ß^c^ (95% CI)
Simple								
Motorized	–		–		–		–	
Combination	1645.0 (131.0 to 199.0)	–	179.0 (147.4 to 210.5)	–	189.2 (156.7 to 221.7)	–	287.1 (234.4 to 339.8)	–
Active	230.6 (187.1 to 273.6)	65.7 (19.2 to 112.1)	267.2 (214.4 to 320.0)	88.2 (35.5 to 141.0)	321.0 (247.6 to 394.3)	131.8 (60.7 to 202.8)	279.4 (154.7 to 404.2)	− 7.6 (− 131.0 to 115.7)
Adjusted^a^								
Motorized	–		–		–			
Combination	161.2 (125.6 to 196.8)	–	173.1 (140.4 to 205.7)	–	184.6 (150.5 to 218.7)	–	241.7 (186.8 to 296.6)	–
Active	215.4 (166.6 to 264.1)	54.2 (5.5 to 102.9)	239.1 (181.9 to 296.3)	66.0 (10.5 to 121.6)	280.1 (197.9 to 362.2)	95.4 (18.3 to 172.6)	266.0 (132.9 to 399.1)	24.3 (− 106.4 to 155.0)

a Adjusted for age, sex, education, employment, ethnicity, income, housing tenure, household car access and children under 16 years of age living at home.

b ‘Motorized’ set as reference category.

c ‘Combination’ set as reference category.

**Table 5:** Linear regression examining the association between time spent in active travel and overall physical activity in respondents (n = 3339) of the iConnect survey conducted in the UK in April 2010.

	n	Mean (SD)	ß (95% CI)	ß^a^ (95% CI)
None	1178	267.7 (344.1)	–	–
0 to 60 min/week	429	267.0 (307.8)	− 0.7 (− 40.8 to 39.4)	− 1.9 (− 42.1 to 38.3)
60 to 150 min/week	685	376.3 (307.8)	108.6 (74.5 to 142.8)	106.2 (71.6 to 140.8)
≥ 150 min/week	1047	661.3 (430.3)	393.6 (363.4 to 423.8)	404.6 (372.6 to 436.6)

a Adjusted for age, sex, education, employment, ethnicity, income, housing tenure, household car access and children under 16 years of age living at home.

## References

[bb0005] Besser L.M., Dannenberg A.L. (2005). Walking to public transit: steps to help meet physical activity recommendations. Am. J. Prev. Med..

[bb0010] Craig C., Marshall A., Sjostrom M. (2003). International Physical Activity Questionnaire: 12-country reliability and validity. Med. Sci. Sports Exerc..

[bb0015] Cummins S., Petticrew M., Higgins C., Findlay A., Sparks L. (2005). Large scale food retailing as an intervention for diet and health: quasi-experimental evaluation of a natural experiment. J. Epidemiol. Community Health.

[bb0020] Curtin R., Presser S., Singer E. (2005). Changes in telephone survey nonresponse over the past quarter century. Public Opin. Q..

[bb0025] Department for Transport (2009). National travel survey: why people travel. London; 2011. http://assets.dft.gov.uk/statistics/releases/national-travel-survey-2010/nts2010-04.pdf.

[bb0030] Department for Transport (2011). National travel survey 2010. London; 2011. http://assets.dft.gov.uk/statistics/releases/national-travel-survey-2010/nts2010-01.pdf.

[bb0035] Goodman A., Guell C., Panter J., Jones A., Ogilvie D. (2012). Healthy travel and the socio-economic structure of car commuting in Cambridge, UK; a mixed-methods analysis. Soc. Sci. Med..

[bb0040] Gordon-Larsen P., Boone-Heinonen J., Sidney S., Sternfeld B., Jacobs D., Lewis C. (2009). Active commuting and cardiovascular disease risk. Arch. Intern. Med..

[bb0045] Hamer M., Chida Y. (2008). Active commuting and cardiovascular risk: a meta-analytic review. Prev. Med..

[bb0050] Hox J.J., De Leeuw E.D. (1994). A comparison of nonresponse in mail, telephone, and face-to-face surveys. Qual. Quant..

[bb0065] Lindström M. (2008). Means of transportation to work and overweight and obesity: a population-based study in southern Sweden. Prev. Med..

[bb0055] Morabia A., Amstislavski P.N., Mirer F.E. (2009). Air pollution and activity during transportation by car, subway, and walking. Am. J. Prev. Med..

[bb0060] Morabia A., Mirer F.E., Amstislavski T.M. (2010). Potential health impact of switching from car to public transportation when commuting to work. Am. J. Public Health.

[bb0070] Nicolaas G. (2004). The use of incentives to motivate hard to get households in National Travel Surveys. Surv. Methods Newsl..

[bb0135] Office of National Statistics Neighbourhood Statistics. http://www.neighbourhood.statistics.gov.uk/dissemination/LeadHome.do.

[bb0100] Ogilvie D., Mitchell R., Mutrie N., Petticrew M., Platt S. (2008). Personal and environmental correlates of active travel and physical activity in a deprived urban population. Int. J. Behav. Nutr. Phys. Act..

[bb0095] Ogilvie D., Bull F.C.L., Powell J. (2011). An applied ecological framework for evaluating infrastructure to promote walking and cycling. Am. J. Public Health.

[bb0090] Ogilvie D., Bull F., Cooper A. (2012). Evaluating the travel, physical activity and carbon impacts of a ‘natural experiment’ in the provision of new walking and cycling infrastructure: methods for the core module of the iConnect study. BMJ Open.

[bb9000] Sahlqvist S., Song Y., Bull F., Adams A., Preston J., Ogilvie D. (2011). Effect of questionnaire length, personalisation and reminder type on response rate to a complex postal survey: a randomised controlled trial. BMC Med. Res. Meth..

[bb0105] Shepard R. (2008). Is active commuting the answer to population health?. Sports Med..

[bb0110] Sisson S.B., Tudor-Locke C. (2008). Comparison of cyclists' and motorists' utilitarian physical activity at an urban university. Prev. Med..

[bb0115] The Information Centre for Health and Social Care (2009). Health Survey for England 2008—Physical Activity and Fitness Summary of Key Findings.

[bb0125] Wen L.M., Rissel C. (2008). Inverse associations between cycling to work, public transport, and overweight and obesity: findings from a population based study in Australia. Prev. Med..

[bb0120] Wen L.M., Orr N., Millett C., Rissel C. (2006). Driving to work and overweight and obesity: findings from 2003 New South Wales Health Survey, Australia. Int. J. Obes..

[bb0130] Wener R.E., Evans M.F. (2007). A morning stroll: levels of physical activity in car and mass transit commuting. Environ. Behav..

